# The Inhibitory Effect of Botulinum Toxin Type A on Rat Pyloric Smooth Muscle Contractile Response to Substance P *In Vitro*

**DOI:** 10.3390/toxins7104143

**Published:** 2015-10-15

**Authors:** Yu-Feng Shao, Jun-Fan Xie, Yin-Xiang Ren, Can Wang, Xiang-Pan Kong, Xiao-Jian Zong, Lin-Lan Fan, Yi-Ping Hou

**Affiliations:** 1 Department of Neuroscience, Anatomy, Histology and Embryology, Key Laboratory of Preclinical Study for New Drugs of Gansu Province, School of Basic Medical Sciences, Lanzhou University, 199 Donggang Xi Road, Lanzhou 730000, China; E-Mails: shaoyf@lzu.edu.cn (Y.-F.S.); xiejfan@163.com (J.-F.X.); renyx@lzu.edu.cn (Y.-X.R.); wangc2012@lzu.cn (C.W.); kongxp530@163.com (X.-P.K.); 2 Department of Human Anatomy, School of Medicine, Hunan Normal University, 371 Tongzipo Road, Changsha 410013, China; 3 Department of Functional Examination, the 2nd Hospital of Gansu Province, Lanzhou 730000, China; E-Mail: zxj0547@163.com; 4 Experimental Center of Medicine, School of Basic Medical Sciences, Lanzhou University, 199 Donggang Xi Road, Lanzhou 730000, China; E-Mail: fanll@lzu.edu.cn

**Keywords:** botulinum toxin type A, substance P, electric field stimulation, neurokinin 1 receptor, antagonist of neurokinin 1 receptor, pyloric smooth muscle contractility, rat

## Abstract

A decrease in pyloric myoelectrical activity and pyloric substance P (SP) content following intrasphincteric injection of botulinum toxin type A (BTX-A) in free move rats have been demonstrated in our previous studies. The aim of the present study was to investigate the inhibitory effect of BTX-A on rat pyloric muscle contractile response to SP *in vitro* and the distributions of SP and neurokinin 1 receptor (NK1R) immunoreactive (IR) cells and fibers within pylorus. After treatment with atropine, BTX-A (10 U/mL), similar to [D-Arg^1^, D-Phe^5^, D-Trp^7,9^, Leu^11^]-SP (APTL-SP, 1 μmol/L) which is an NK1R antagonist, decreased electric field stimulation (EFS)-induced contractile tension and frequency, whereas, subsequent administration of APTL-SP did not act on contractility. Incubation with BTX-A at 4 and 10 U/mL for 4 h respectively decreased SP (1 μmol/L)-induced contractions by 26.64% ± 5.12% and 74.92% ± 3.62%. SP-IR fibers and NK1R-IR cells both located within pylorus including mucosa and circular muscle layer. However, fewer SP-fibers were observed in pylorus treated with BTX-A (10 U/mL). In conclusion, BTX-A inhibits SP release from enteric terminals in pylorus and EFS-induced contractile responses when muscarinic cholinergic receptors are blocked by atropine. In addition, BTX-A concentration- and time-dependently directly inhibits SP-induced pyloric smooth muscle contractility.

## 1. Introduction

Botulinum toxin (BTX) is produced from the bacterium *clostridium botulinum*. Seven distinct serotypes have been identified and designated as types A, B, C, D, E, F and G [1,2,3]. It is well investigated that the major target of BTX is the cholinergic nerve ending of neuromuscular junctions in skeletal muscles, where the inhibition of acetylcholine (ACh) release results in neuromuscular blockade and paralysis [4,5]. Currently, BTX-A has been successfully used in the treatment of voluntary muscle contraction disorders such as strabismus, dystonia, and tremors [4,6,7,8,9].

However, in smooth muscles, an injection of BTX-A into pylorus in patients with gastroparesis might relax the pylorus and facilitate gastric emptying. Several small open-label studies have also shown this in diabetic gastroparesis [10,11] and in idiopathic gastroparesis [12]. In gastrointestinal smooth muscle, BTX-A appears to reduce cholinergic transmission by inhibiting ACh release, as shown *in vitro* [13,14] and *in vivo* [15]. In addition, our recent studies have demonstrated that BTX-A intrasphincteric injection through guide cannula in free move rats dose-dependently caused an inhibition of slow wave in amplitude but not in frequency, a diminishment of spike activity in amplitude and spike burst of pyloric myoelectrical activity, and a reduction of substance P (SP) content within pylorus [16]. These data suggest that BTX-A inhibit not only the ACh release but also SP release from the autonomic and enteric nervous terminals in pylorus.

SP, a major excitatory non-cholinergic neurotransmitter, depolarizes the membrane potential and thus induces contraction in gastrointestinal smooth muscle [17,18]. It is an undecapeptide belonging to the tachykinin family and can induce strong contractions in pylorus via neurokinin 1 receptor (NK1R), the preferred receptor for SP [19]. However, no experimental evidence that BTX-A inhibits the pyloric contractile response induced by SP released from nervous terminals or SP dosed has been documented so far.

The present studies were designed to provide information regarding the inhibitory target of BTX-A for SP. Primarily, to investigate the inhibitory effect of BTX-A on contractile tension and frequency of pyloric muscle strips after the effect of ACh being abolished by atropine, an antagonist of cholinergic muscarinic receptor, and compare with the inhibitory effect of NK1R antagonist in same condition. Secondly, measure the pyloric smooth muscle contractile response to SP under a range concentration of BTX-A incubation. Finally, examine the distributions of SP and NK1R immunoreacive cells and fibers in pylorus treated without and with BTX-A by immunofluorescence.

## 2. Results

### 2.1. Inhibitory Effect of BTX-A on Contractile Response to SP Release from Pylorus

**Figure 1:**
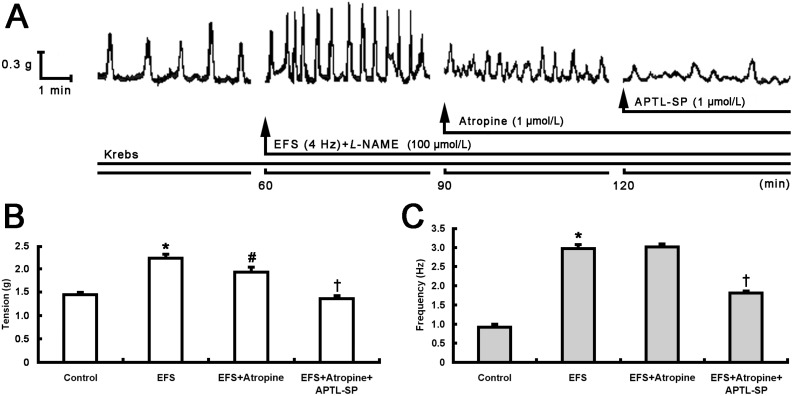
Effect of APTL-SP on EFS-induced pyloric contractile responses following atropine action. (**A**) Representative 2-h contractile recording curves show that EFS (4 Hz) enhanced spontaneous contraction of pyloric smooth muscle strip incubated with Krebs solution containing Nω-nitro-L-arginine methyl ester (L-NAME, 100 μmol/L), then atropine (1 μmol/L) addition at 90 min partially decreased the EFS-induced contractile responses and subsequent addition of APTL-SP (1 μmol/L) at 120 min further decreased the contractility. The statistical significance in contractile tension (**B**) and frequency (**C**) of pyloric strips performed respectively with Krebs solution as control, EFS, EFS + Atropine and EFS + Atropine + APTL-SP are shown. Data are expressed as means ± SEM, *n* = 8, analyzed by one-way ANOVA followed by Fisher’s PLSD test. * *p* < 0.001, EFS *vs.* control; ^#^ *p* < 0.005, EFS + Atropine *vs.* EFS; ^†^ *p* < 0.001, EFS + Atropine + APTL-SP *vs.* EFS + Atropine.

Electric field stimulation (EFS) was used to induce neurotransmitter release from pylorus and increase pyloric smooth muscle contractility. In the first group, as shown in Figure 1, EFS significantly induced an increase in pyloric contractile tension (from 1.458 ± 0.029 to 2.240 ± 0.070 g, *p* < 0.001; Figure 1A,B) and frequency (from 0.928 ± 0.038 to 2.980 ± 0.078 Hz, *p* < 0.001; Figure 1A,C). The addition of atropine (1 μmol/L) after 30 min partially decreased contractile tension (from 2.240 ± 0.070 to 1.944 ± 0.090 g, *p* < 0.005), but did not affect its frequency (Figure 1A–C). Subsequently, [D-Arg1, D-Phe5, D-Trp7,9, Leu11]-SP (APTL-SP) was added and it further decreased contractile tension (from 1.944 ± 0.090 to 1.376 ± 0.036 g, *p* < 0.001) and frequency (from 3.028 ± 0.054 to 1.820 ± 0.034 Hz, *p* < 0.001) (Figure 1A–C).

**Figure 2:**
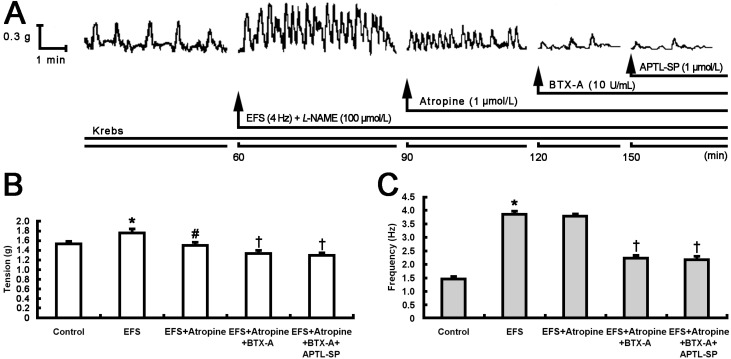
Effect of BTX-A on EFS-induced pyloric contractile responses following atropine action. (**A**) Representative 2.5-h contractile recording curves show that EFS (4 Hz) enhanced spontaneous contraction of pyloric smooth muscle strip incubated with Krebs solution containing L-NAME (100 μmol/L), then atropine (1 μmol/L) addition at 90 min partially decreased the EFS-induced contractile responses, subsequent addition of BTX-A (10 U/mL) at 120 min further decreased the contractility and finally, APTL-SP (1 μmol/L) addition at 150 min did not act on. The statistical significance in contractile tension (**B**) and frequency (**C**) of pyloric strips performed respectively with Krebs solution as control, EFS, EFS + Atropine, EFS + Atropine + BTX-A and EFS + Atropine + BTX-A + APTL-SP are shown. Data are expressed as means ± SEM, *n* = 8, analyzed by one-way ANOVA followed by Fisher’s PLSD test. * *p* < 0.001, EFS *vs.* control; ^#^ *p* < 0.001, EFS + Atropine *vs.* EFS; ^†^ *p* < 0.001, EFS + Atropine + BTX-A or EFS + Atropine + BTX-A + APTL-SP *vs.* EFS + Atropine.

In the second group (Figure 2), BTX-A (10 U/mL) instead of APTL-SP further decreased contractile tension and frequency (Figure 2A–C) following atropine. The inhibitory effect of BTX-A on pyloric contractility was similar to that of APTL-SP. Noticeably, subsequent addition of APTL-SP following BTX-A action had no effect on pyloric contractility in tension and frequency (Figure 2A–C).

### 2.2. Inhibitory Effect of BTX-A on SP-induced Contractile Response

**Figure 3:**
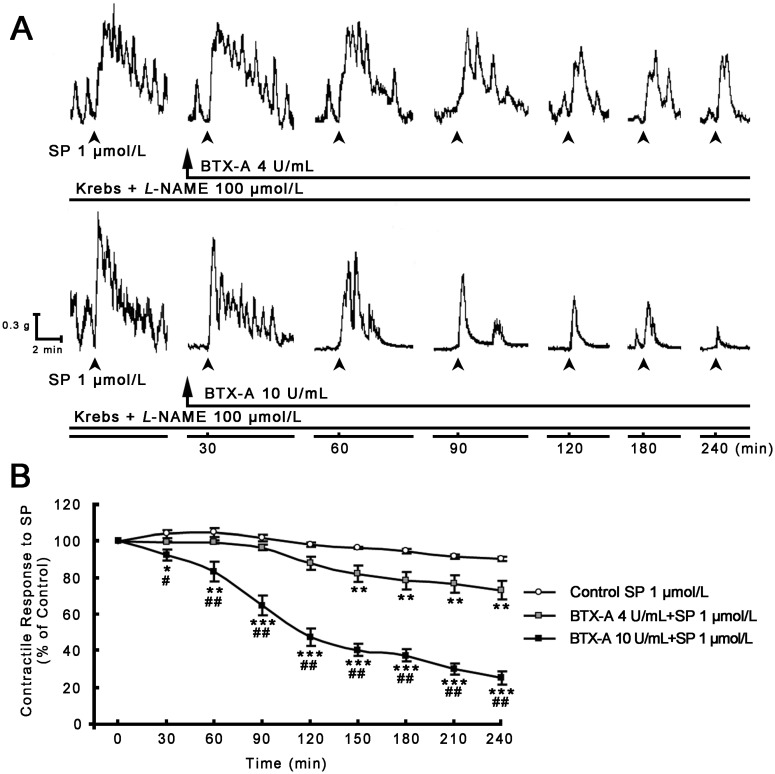
Effect of BTX-A on SP-induced pyloric contractile responses. (**A**) Representative 4-h contractile recording curves show that SP induced an increase in pyloric spontaneous contractions, and that incubation with BTX-A from 4 (upper panel) to 10 U/mL (lower panel) concentration- and time-dependently decreased SP-induced pyloric contractile responses (SP was added at interval 30 min and shown by head of arrow); (**B**) BTX-A statistically inhibited SP-induced contractile responses with concentration- and time-dependent manner. Data are expressed as means ± SEM, *n* = 8 in each group, analyzed by one-way ANOVA followed by Fisher’s PLSD test. * *p* < 0.05, ** *p* < 0.01, *** *p* < 0.001, *vs.* control; ^#^ *p* < 0.01, ^##^ *p* < 0.001, BTX-A 10 U/mL *vs.* 4 U/mL.

The inhibitory effect of different concentrations of BTX-A on SP-induced pyloric muscle contractility was further studied. As shown in Figure 3, SP (1 μmol/L) induced an increase in pyloric contractility (Figure 3A), whereas pyloric muscle strips incubated with 4 and 10 U/mL BTX-A concentration- and time-dependently decreased the contractile response to SP through the experimental period (Figure 3A,B), and respectively decreased SP-induced contractions by 26.64% ± 5.12% (*p* < 0.01) and 74.92% ± 3.62% (*p* < 0.001) at the end of 4 h (Figure 3B).

### 2.3. Distribution of SP- and NK1R-Immunoreactive Expressions in Pylorus without or with BTX-A Treatment

**Figure 4:**
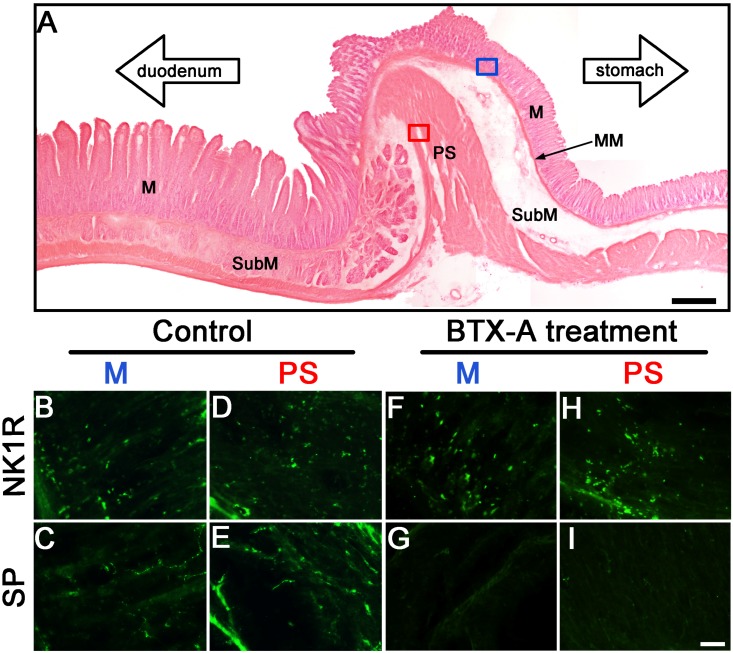
Distributions of SP- and NK1R-IR fibers and cells within pylorus treated without or with BTX-A. (**A**) Photograph shows the structure of pyloric longitudinal tissue stained with HE: M, mucous; MM, muscularis mucosae; PS, pyloric sphincter; SubM, submucos. Bar = 500 μm. (**B**–**I**) Photographs show the distributions and morphological characteristics of NK1R- and SP-IR cells and fibers in mucous (blue box in **A**) and circular muscles (red box in **A**) without (control, **B**–**E**) or with BTX-A treatment (**F**–**I**). NK1R-IR expressed cells mainly in lamina propria of mucous and scattered in epithelium of mucous (**B**) and diffusely expressed in circular muscle (**D**). Varicosities of SP-IR fibers vertically projected from lamina propria to epithelium of mucosa (**C**) and scattered in circular muscle (**E**). After 10 U/mL BTX-A treatment, a dense expression of NK1R-IR cells was found in M (**F**) and PS (**H**). However, the SP-IR fibers were few in M (**G**) and PS (**I**). Bar = 25 μm.

In the pylorus without BTX-A treatment, beaded SP immunereactive (IR) fibers located in lamina propria and were preference near to epithelium of mucosa (Figure 4C). NK1R-IR cells were mainly found in lamina propria, and scattered in epithelium of mucosa (Figure 4B). In pyloric sphincter, which composed by thickening circular muscle, SP-IR fibers (Figure 4E) and NK1R-IR cells (Figure 4D) were diffusely expressed in these areas. In the pylorus treated with BTX-A, the expression of NK1R-IR cells in mucous (Figure 4F) and pyloric sphincter (Figure 4H) was similar to the pylorus treated without BTX-A. However, fewer SP-IR fibers were observed in mucous (Figure 4G) and pyloric sphincter (Figure 4I) compared to control. The fact that both NK1R-IR and SP-IR expressed in the same area indicates that SP through NK1R acts on pyloric smooth muscle. BTX-A inhibited SP release form enteric terminals in pylorus.

## 3. Discussion

Intrasphincteric injection of BTX-A has been recently proposed as an alternative to treat pyloric dysfunction or pylorospasm [14] and demonstrated that it induces a decrease in pyloric myoelectrical activity and pyloric SP content in free move rats [16]. Our current results documented that atropine and subsequent APTL-SP incrementally decreased EFS-induced contractile responses in rat pyloric smooth muscle strips (Figure 1). When BTX-A instead of APTL-SP was added following atropine, its inhibitory effect on EFS-induced contractile responses was similar to APTL-SP, and then APTL-SP addition did not influence contractile responses to EFS again (Figure 2). EFS is a perfect method to induce neurotransmitters release including cholinergic and non-cholinergic neurotransmitters in gastrointestine [14,20]. The evidence of APTL-SP, an NK1R antagonist, further inhibited EFS-induced contractility after atropine blocking muscarinic cholinergic receptors suggests that EFS does not induce only ACh, but also SP release from pylorus. By comparison, the evidence that APTL-SP administration does not act on contractile responses to EFS following inhibition of BTX-A indicates that BTX-A prevents presynaptic vesicles containing SP from fusion with plasma membrane and leads to SP inaction on NK1R within pylorus. SP, similar to ACh can induce strong contractions in pylorus [19] and enhance spike discharges in gastric myoelectrical activity [21]. Several experimental studies have also demonstrated that BTX-A inhibits SP release from pyloric sphincter in rats [16], nasal mucosa of rat allergic rhinitis [22], trigeminal nerve terminals of the rabbit iris sphincter [23], cultured embryonic dorsal root ganglion neurons [24] and isolated bladders in rat bladder models of both acute injury and chronic inflammation [25].

Our results have further shown that BTX-A concentration- and time-dependently inhibited SP-induced contractile tension and frequency in pylorus (Figure 3). When incubating of the muscle strips to BTX-A for a prolonged period (4 h), the inhibitory effects of BTX-A on SP-induced contractions were not immediate; the onset of inhibitory effect was gradual and slowly progressive. This time-dependent effect may be related to either the mechanism of action of BTX-A or diffusion of the toxin into the tissue. When incubation of the muscle strips at four and 10 U/mL BTX-A respectively decreased the contractile response to SP by 26.64% ± 5.12% and 74.92% ± 3.62% after 4 h suggesting its inhibitory effects on SP-induced pyloric contractility were also concentration-dependent. The inhibitory characteristics of concentration- and time-dependent effects of BTX-A have also been documented on pyloric myoelectrical activity *in vivo* [16], pyloric and antral contractility *in vitro* [14], common bile duct (CBD) pressure [15,26] and lipogenesis [27].

Besides, our study has examined that the distribution of neuronal SP- and NK1R-containing cells and nerve fibers in rat pylorus for understanding the targets of BTX-A action. SP-IR fibers and NK1R-IR cells located in mucosa and circular muscle layers of pylorus (Figure 4). However, fewer SP-IR fibers in the pylorus treated with BTX-A were observed in mucous (Figure 4G) and pyloric sphincter (Figure 4I) compared to control. These results indicate that SP through NK1R innervates inner edge of the circular muscle and layer adjacent to the mucosa of pylorus, which plays an important role in the regulation of gastric emptying [28,29,30].

**Figure 5:**
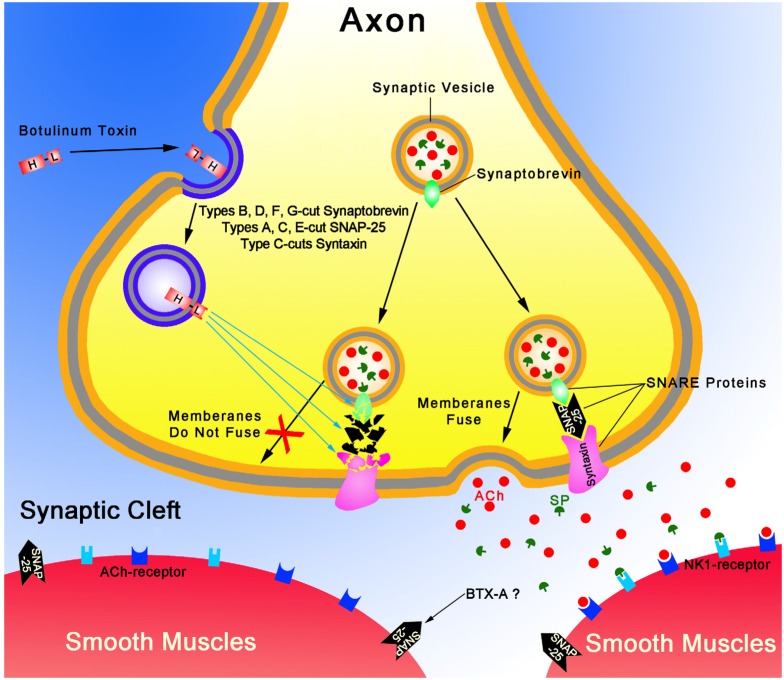
A presumed mechanism of inhibitory effect of BTX-A on smooth muscles is schematized.

Based on the current and previous studies, we proposed the mechanism of inhibitory effects of BTX-A on smooth muscle (Figure 5). Classically, in striated muscle, BTX-A inhibits ACh release from cholinergic nerves [4]. A four-step mechanism consisting of binding, internalization, translocation and cleaving soluble NSF accessory protein receptor (SNARE) protein is currently the accepted view to explain BTX-A intoxication [31,32,33,34]. BTX-A consists of a heavy chain (HC, ~100 kDa) and a light chain (LC, ~50 kDa) linked by a single disulfide bound and non-covalent forces [35,36]. HC can bind to the synaptic membrane and then its entire molecule is internalized into the synaptic terminal by receptor-mediated endocytosis. LC selectively cleaves the synaptosomal-associated protein of 25 kDa (SNAP-25) [37], leading to the ACh-containing vesicles can no longer fuse with the plasma membrane, and exocytosis of ACh is inhibited [38,39,40]. In gastrointestinal smooth muscle, SP coexists with ACh and enkephalin [41,42,43,44,45], thus, BTX-A may inhibit ACh release accompanied with the inhibition of SP release (Figure 5). SP, in this study, may acts primarily on NK1R to contract pyloric smooth muscles, because the contractions to EFS were further inhibited by APTL-SP, an NK1R antagonist, when atropine blocked muscarinic cholinergic receptors. BTX-A directly inhibits exogenous SP-induced smooth muscle contractility as evidenced by the time-dependently abolished contractile response to SP administration. The effect of BTX-A might directly involve in acetylcholine related contractility which results in a low response to SP administration, since SP as a co-neurotransmitter that appears to be important for the maintenance of muscular responsiveness to the principal excitatory neurotransmitter, ACh [46]. The exact cellular mechanism of BTX-A inhibiting SP-induced contractions was not detailed in the current study. Noticeably, SNAP-25, the substrate for BTX-A, has also been found in gastrointestinal smooth muscle and inhibits outward potassium currents under physiologic condition. When the inhibition of SNAP-25 removed by BTX-A, it leads the membrane hyperpolarized and thereby muscle tone decreased [47,48]. This suggests that besides the classical presynaptic neuron sites to reduce neurotransmitter release, there is also another site for BTX-A in regulating muscle contractility.

In summary, the evidence that SP- and NK1R-IR fibers and neurons located within mucosa and circular muscle layer of pylorus suggests that SP through NK1R innervates pyloric muscle for regulation of gastric emptying. BTX-A reduced the expression of SP-IR fibers in pylorus, and further inhibits EFS-induced contractile responses after atropine blocking muscarinic cholinergic receptors, and then NK1R antagonist administration does not act on EFS-induced contractile responses, suggesting inhibition of SP release from pyloric nervous terminals. BTX-A directly inhibits SP-induced pyloric smooth muscle contractility in a concentration- and time-dependent manner.

## 4. Materials and Methods

### 4.1. Animals

Adult Sprague–Dawley rats (Experimental Animal Center, Lanzhou University, Lanzhou, China) weighing 200–250 g were housed individually in cage with food and water ad libitum, kept on a 12-h light-dark cycle (light 07:00–19:00 h), room temperature at 21 ± 1 °C and relative humidity at 50% for seven days before experiments. Experimental procedures were carried out in accordance with European Communities Council Directive of 24 November 1986 (86/609/EEC) and approved by the Institutional Animal Care and Use Committees of Gansu Province Medical Animal Center and Lanzhou University. All performances were undergone to minimize animal suffering and to use only the number of animals necessary to produce reliable scientific data.

### 4.2. Pyloric Muscle Strip Preparation

Before pyloric muscle strip preparation, animals were fasted for 18 to 24 h, but water *ad libitum*. An approximately 10 mm × 2 mm pyloric circular muscle strip was rapidly separated from per stomach after rat sacrificed by CO_2_ asphyxiation. The isolated strip was carefully rinsed and suspended by a string in an incubation bath containing 5 mL Krebs bicarbonate buffer solution composed (in mmol/L): 118 NaCl, 4.7 KCl, 2.5 CaCl_2_, 1.2 MgSO_4_, 24.9 NaHCO_3_, 1.2 NaH_2_PO_4_ and 12.2 glucose, in which pH was adjusted to 7.4, constant temperature was maintained at 37 °C and oxygenation was constantly bubbled with 95% O_2_-5% CO_2_. One terminal of strip was attached to a muscular force transducer (JH-2, Space Medico-engineering Institute, Beijing, China) connected to a Mac Lab (model BL-420E, TM, Chengdu, China) for isometric tension recording. Two platinum electrodes placed adjacent and parallel to the long axis of the pyloric muscle strip, connected with electric stimulator (model Biolap 420E) for setting EFS (parameter with 100 V, 4 Hz, 0.5 ms pulse width duration). Muscle strips were allowed to equilibrate for 60 min under a basal loading tension of 1 g, and its spontaneous contractile waves were regularly emerged as its own control before EFS and adding drugs.

### 4.3. Experimental Protocols

In order to compare the inhibitory effect of BTX-A with antagonist of NK1R on pyloric muscle which contraction-induced by SP released from nervous terminals, after control response to EFS plus 100 μmol/L Nω-nitro-L-arginine methyl ester (L-NAME, Sigma, St. Louis, MO, USA), an inhibitor of nitric oxide-mediated relaxation, for 30 min, 1 μmol/L atropine (Sigma, St. Louis, MO, USA), an antagonist of cholinergic muscarinic receptor, and 1 μmol/L APTL-SP (Sigma, St. Louis, MO, USA), an NK1R antagonist, were respectively added at intervals of 30 min in the first group of muscle strips (*n* = 8, Figure 1). In the second group (*n* = 8), after control response to EFS + L-NAME, atropine, 10 U/mL BTX-A (GMP Nos. S10970037, 20061001, Lanzhou Institute of Biological Products, Lanzhou, China) and APTL-SP (1 μmol/L) were respectively added at intervals of 30 min (Figure 2).

To determine the inhibitory effect of BTX-A on SP-induced pyloric muscle contractions, after initial control response to SP (1 μmol/L Sigma, St. Louis, MO, USA) for 30 min, incubational solutions were respectively replaced by Krebs + L-NAME 100 μmol/L + 4 or 10 U/mL BTX-A (each group *n* = 8) and continued for 4 h. SP (1 μmol/L) was added at intervals of 30 min during the period (Figure 3).

### 4.4. Identification for the Distribution of SP and NK1R Cells and Fibers in Pylorus

Under deep anesthesia with chloral hydrate (400 mg/kg body mass, i.p.), five adult rats without BTX-A treatment as control were perfused through the ascending aorta with 100 mL of saline, followed by 350 mL of phosphate buffer (PB, 0.1 mol/L, pH 7.4, 4 °C) containing 4% paraformaldehyde. After perfusion, pylorus was carefully dissected from stomach and post-fixed overnight in the same solution. Pyloric muscle strips from 5 adult rats treated with BTX-A were prepared as described before, and incubated into Krebs solution containing 10 U/mL BTX-A at 37 °C for four hours, then into the 4% paraformaldehyde to post-fixed overnight. All pylorus were rinsing for 48–72 h in PB containing 30% sucrose (4 °C), and sectioned (30 μm) on a cryostat at −25 °C. Some sections were performed with immunofluorescence procedure as described in our previous studies [16,49]. Briefly, adjacent sections were respectively incubated with rabbit anti-NK1R (Millipore, Temecula, CA, USA) or rabbit anti-SP (Millipore, Temecula, CA, USA) serum diluted 1:2000 in 0.01 M PB saline containing 0.25% Triton X-100 (PBS-T, pH 7.4) for 24 h on an agitator at 4 °C. After rinses, the sections were placed in fluorescein isothiocyanate (FITC)-conjugated goat anti-rabbit IgG (1:500; Invitrogen, Carlsbad, CA, USA) for 2 h at 37 °C. Parallel control series were treated in the absence of the primary antibodies and in the presence of normal rabbit sera to assure the absence of any non-specific labeling with these procedures. Sections were mounted and then coverslipped with glycerol. Fluorescent immunostained cells and fibers were visualized by fluorescence microscope (Nikon, Tokyo, Japan). Other sections were mounted on gelatin-coated glass slides, stained with hematoxylin and eosin (HE) for histological assessing the distribution and morphology of SP and NK1R containing cells. Digital images were optimized for image resolution in Adobe Photoshop 12.0.

### 4.5. Data Analysis

Each preparation served as its own control with the tension and frequency of muscle stripe contractility in Krebs solution was compared respectively with the contractile response to EFS or drugs. The values were expressed as means ± SEM. The data were statistically analyzed using one-way ANOVA followed by Fisher’s PLSD test. Differences between means from experimental groups were considered significant at the *p* < 0.05.

The morphological distributions of NK1R- and SP-IR cells and fibers in pyloric mucous and sphincter (Figure 4A) from all samples treated with or without BTX-A were analyzed using a 490 nm excitation filter and 520 nm emission filter under fluorescence microscope (Nikon). The NK1R- and SP-IR cells and fibers were determined according to Harrington *et al.* [50] and Lomax *et al.* [51], in which SP-IR fibers show a beaded fluorescence and distribute similar to enteric nervous plexus, and NK1R-IR cellular fluorescence locate within mucous and sphincter.
